# Effect of extra-amniotic Foley’s catheter and vaginal misoprostol versus vaginal misoprostol alone on cervical ripening and induction of labor in Kenya, a randomized controlled trial

**DOI:** 10.1186/s12884-018-1793-2

**Published:** 2018-07-12

**Authors:** Alfred Osoti, Davies Kiprop Kibii, Tito Mario Kual Tong, Innocent Maranga

**Affiliations:** Department of Obstetrics and Gynaecology, University of Nairobi, P.O. Box 19676, Nairobi, 00202 KNH Kenya

**Keywords:** Failed induction, RCT, Foley’s, Misoprostol, Induction-to-delivery

## Abstract

**Background:**

The safest, most effective and fastest combined approaches to induction of labor is unknown. In an open-label randomized clinical trial we evaluated the efficacy of combination of extra-amniotic Foley’s catheter and vaginal misoprostol compared to vaginal misoprostol alone for cervical ripening and induction of labor on the incidence of failed induction, induction-to-delivery interval and adverse maternal and perinatal outcomes.

**Methods:**

Pregnant women at gestational age of 28 weeks or greater admitted at Kenyatta National Hospital, Kenya for induction of labor were enrolled then randomized to either a combination of extra-amniotic Foley’s catheter inflated by 30 cm^3^ of normal saline and 25 micrograms of vaginal misoprostol or 25 micrograms of vaginal misoprostol alone. Women underwent 6 hourly reviews and additional misoprostol inserted if required. The primary outcome was incidence of failed induction. Secondary outcomes were induction-to-delivery interval and adverse maternal and perinatal outcomes. We conducted an intent-to-treat analysis and compared means or medians using t-test or Wilcoxon rank, proportions using Chi-square or Fishers test as appropriate. Induction-to-delivery interval were compared using the log-rank test. *P*-values of < 0.05 and 95% confidence intervals that excluded the null were considered statistically significant.

**Results:**

Between February and May 2016, we enrolled 180 of 237 pregnant women admitted for induction of labor and randomized them to either a combination of extra-amniotic Foley’s catheter and vaginal misoprostol (*n* = 90) or vaginal misoprostol alone (*n* = 90). The socio-demographic and obstetric characteristics were similar between the two groups. Failed induction rates were lower but not statistically significant following combined extra-amniotic Foley’s catheter and vaginal misoprostol (8.9%) versus vaginal misoprostol alone (11.1%). The mean induction-to-delivery time was 4.8 h shorter in the combined extra-amniotic Foley’s catheter and vaginal misoprostol (mean 18.9, standard deviation (SD) 7.2 h) compared to misoprostol only group (mean 14.1, SD 6.9 h) (log-rank test, *p* < 0.001). Maternal and perinatal complications were similar between the two groups.

**Conclusions:**

Extra-amniotic Foley’s catheter and vaginal misoprostol for cervical ripening and induction of labor did not significantly lower the incidence of failed induction but safely shortened induction-to-delivery time compared to vaginal misoprostol only.

**Trial registration:**

Trial was retrospectively registered on 14–03-2016 PACTR201604001535825.

**Electronic supplementary material:**

The online version of this article (10.1186/s12884-018-1793-2) contains supplementary material, which is available to authorized users.

## Background

Induction of labor, the stimulation of uterine contractions during pregnancy before spontaneous onset of labor in order to accomplish vaginal delivery occurs in about 25% of pregnancies in developed countries and even with higher frequencies in some developing countries [1–3]. Induction of labor is performed for various indications, but primarily when continuation of pregnancy poses a risk to maternal or fetal health. Induction of labor can be achieved by chemical (for example prostaglandins and oxytocin), mechanical (for example extra-amniotic Foley’s catheter with or without normal saline, amniotomy and laminaria) or both [2, 4]. Women with unfavorable cervix or poor Bishop’s score require cervical ripening primarily by synthetic prostaglandin E1 analogue (misoprostol) or E2 (dinoprostone) [4]. In a 2011 survey conducted among 262 women undergoing induction of labor at Kenyatta National Hospital, Kenya, the methods of cervical ripening and labor induction were: 38.5% misoprostol only, 40.5% misoprostol, amniotomy and oxytocin, 8% dinoprostone, amniotomy and oxytocin, 8.8% oxytocin, 4.2% dinoprostone alone [5]. In Kenya, combined mechanical and chemical methods are not routinely used either serially or concurrently.

Although various safe methods for labor induction are available, the most effective and fastest approaches to inducing labor is unknown, necessitating the combination of different approaches [1, 6, 7]. In addition, the most effective method in reducing failed induction of labor, the inability to achieve more than 3 cm cervical dilatation after 24 h of induction of labor has not been examined in Kenya. Prior studies reported either no difference or a shorter mean induction-to-delivery interval comparing combination of extra-amniotic Foley’s catheter and vaginal misoprostol versus vaginal misoprostol alone [6–11]. However, these studies did not report on failed induction as a primary outcome. In a four-arm randomized trial, various combination methods reduced median time-to-delivery compared to a single-agent method by an average of 4 h [7]. In this study, the hazards of delivery for single-agent method misoprostol–cervical Foley’s was doubled by misoprostol–cervical Foley’s. Although a secondary outcome, failed induction did not vary across the four groups.

Failed induction of labor and prolonged induction-to-delivery time are associated with increased hospitalization costs, maternal and parental anxiety and are likely to increase adverse maternal and perinatal outcomes when safe and timely vaginal delivery is not realized [12]. The potential effect and safety of combination of pharmacological (misoprostol) and mechanical methods (extra-amniotic Foley’s catheter) in reducing failed induction of labor in Kenya has not been reported. Combined methods may reduce the incidence of failed induction and enhance progress of labor due to the synergistic effect of prostaglandin and mechanical dilatation on cervical ripening and progress of labor [7].

We conducted an open-label randomized clinical trial among women undergoing cervical ripening and induction of labor to evaluate the effect of combined extra-amniotic Foley’s catheter and vaginal misoprostol compared to vaginal misoprostol alone on incidence of failed induction, induction-to-delivery interval and adverse maternal and perinatal outcomes in Kenya.

## Methods

### Study setting

This was a two-arm open label randomized clinical trial conducted at the Kenyatta Hospital (KNH) antenatal and labor wards. As the largest and oldest national teaching and referral hospital in Kenya, KNH receives patients from Nairobi, the capital city, its environs as well as referrals from other hospitals in Kenya. The methods of cervical ripening in this setting are single-agent either as prostaglandins (misoprostol or dinoprostone) or Foley’s catheter and often these methods are not combined. A busy hospital, KNH conducts an estimated 10,000 deliveries per year.

### Study participants

The study population comprised pregnant women admitted for induction of labor at a gestational age of 28 weeks or greater for any of the following indications; late term or post term pregnancies, pre-eclampsia, chronic hypertension, gestational diabetes, oligohydramnios, or intrauterine fetal demise. Induction of labor was defined as the stimulation of uterine contractions during pregnancy before spontaneous onset of labor due to fetal or maternal health concerns. Women were consecutively sampled, screened for eligibility and enrolled if they had singleton cephalic pregnancies, intact membranes, Bishop’s score less than 6, and no contraindications for vaginal delivery after providing informed consent [13]. We excluded women who had favorable Bishop’s score, fetal growth restriction, previous cesarean sections or other uterine surgeries, multiple gestation, contraindication to prostaglandins, fetal anomalies, estimated fetal weight more than 4000 g, placenta previa, non-reassuring fetal status, grand multiparity, HIV infection and uncertain gestational age.

### Participant recruitment

The study population were pregnant women scheduled for induction of labor and admitted to the labor ward either from home or the antenatal clinics. As routinely practiced, the team leader, the consultant obstetrician(s) covering labor ward or the antenatal wards made the decisions for cervical ripening and induction of labor. Trained research assistants who were postgraduate students in Obstetrics and Gynecology, approached the women for screening and obtained informed consent prior to conducting any study procedures.

### Ethical approval

The Department of Obstetrics and Gynecology of the University of Nairobi (UoN) and the KNH/UoN Ethical Research Committee (P71/02/2015) approved the study. The study was registered at the Pan African Clinical Trials Registry (PACTR201604001535825) (Additional file 1, study protocol file) [13]. The extra-amniotic Foley’s catheter catheters were bought by the funding agency, KNH Research and Programs Department. This study was subject to monitoring by an independent Data Safety and Monitoring Board (DSMB). Interim analyses for efficacy and/or effectiveness were conducted when 50 and 75% of participants had completed follow-up. The study was allowed to continue as there was no obvious harm or benefit established and there was no multiplicity adjustment. Adverse events were reported to the DSMB and the KNH/UoN Ethical Review Committee.

### Randomization and blinding

Randomization scheme was created by an independent statistician in blocks of 10 and ratio of 1:1 using a computer generated random sequence. Randomization number and study arm were kept in sequentially serially numbered opaque envelopes. Each envelope was opened once an eligible participant provided informed consent after which the assigned treatment was administered. The participants, research assistants including outcome assessors were aware of the treatment allocation at the time of assignment of treatment, monitoring or assessment of outcomes due to the nature of the intervention and therefore were not blinded. However, the study statistician was unaware of the study arms.

### Interventions

Induction of labor was defined as the stimulation of uterine contractions during pregnancy before spontaneous onset of labor in order to accomplish vaginal delivery. Since unfavorable cervix is ripened prior to initiation of uterine contractions, the interventions are typically for both cervical ripening and induction of labor. Cervical ripening was defined as cervical remodeling to facilitate cervical softening, thinning, and dilation.

Pregnant women who were assigned to the misoprostol alone arm received 25 mcg of misoprostol inserted into the posterior fornix 6 hourly up to a maximum of 4 doses, Bishop’s score more than 6 or active labor. If required amniotomy and augmentation of labor with oxytocin were conducted at the discretion of the team providing intrapartum care. In the combination arm, an 18-French extra-amniotic Foley’s catheter with a 30 cm^3^ balloon was placed just above the internal cervical os and then inflated with 30 cm^3^ of sterile water for injection. The tip of the length of the extra-amniotic Foley’s catheter was strapped to the subject’s inner right thigh under slight tension so that the balloon could exert pressure on the cervical os. At the same time 25 mcg of misoprostol was inserted into the posterior fornix. If unsuccessful at first attempt, extra-amniotic Foley’s catheter insertion was attempted every 6 h together with subsequent 6 hourly doses of 25 mcg of misoprostol until Bishop’s score was greater than 6, 6 doses of misoprostol were administered, rupture of membranes or active labor occurred. When extra-amniotic Foley’s catheter fell off, labor was augmented or amniotomy performed as per the recommendations of the intrapartum care team. In each arm, women were examined every 6 h.

### Outcomes

The primary outcome of the study was incidence of failed induction, defined as inability to achieve cervical dilatation of more than 3 cm 24 h after initiation of induction. Secondary outcomes were time from onset of induction to delivery in hours (induction-to-delivery time) and incidence of adverse maternal outcomes like postpartum hemorrhage, uterine rupture and adverse perinatal outcomes including poor APGAR scores, Neonatal Intensives Care Unit admission and non-reassuring fetal status.

### Sample size and statistical methods

The failed induction rate in a recent Kenyan study was 26% [5]. We postulated that misoprostol and extra-amniotic Foley’s catheter (90% success) compared to misoprostol alone (74% success) would reduce the proportion of failed induction from 26 to 10%. Therefore, we estimated that randomizing 180 pregnant women (90 per group) would provide 80% power to detect the stated difference of 16% in the failed induction rates following combination of extra-amniotic Foley’s catheter with misoprostol versus misoprostol alone, under a two-sided alpha = 0.05 level of significance.

Demographic, clinical and laboratory characteristics, primary and secondary outcomes were compared between the two study arms. Continuous variables were summarized using means (SD) and compared using the two-sample t-test if normality assumptions are met; or summarized using medians and interquartile ranges and compared using nonparametric Wilcoxon rank sum test. Categorical variables were summarized using counts and proportions and compared between study groups using Pearson’s Chi-square (Chi-2) tests or Fisher’s exact tests as appropriate. Induction-to-delivery interval was recorded in hours and survival functions of the two intervention groups compared using the log-rank test in a Kaplan-Meir curve. A 95% Confidence Interval (CI) excluding the null and *p*-level of < 0.05 were considered significant. All analyses were conducted using SPSS® version 21 under intent-to-treat analysis.

### Data collection

Data was collected using questionnaires administered by the principal investigator and four trained research assistants, all postgraduate residents in the Department of Obstetrics and Gynecology, University of Nairobi. Information was obtained from participant interviews, review of medical records and clinical examination. Participant information was stored safely in a password-protected computer and backed up on a dedicated encrypted USB drive. The research assistants together with the principal investigator held training sessions in labor ward on counseling, obtaining informed consent, standardization of speculum examinations, insertion of extra-amniotic Foley’s catheter catheter and vaginal misoprostol.

## Results

**Fig. 1:**
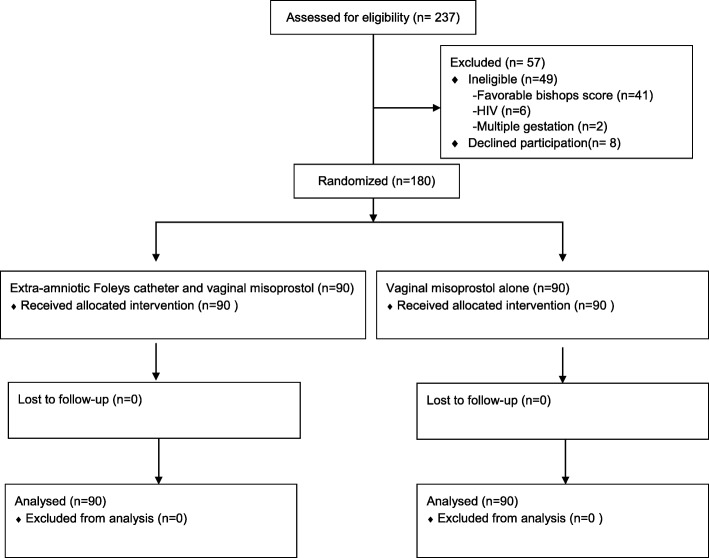
Study participant flow

Between February and May 2016, a total of 180 (76%) of 237 pregnant women who were scheduled for induction of labor were enrolled (Fig. 1). Of the 57 who were excluded: 41 (72%) had favorable Bishop’s score, 8 (14%) refused to participate, 6 (11%) were HIV infected and 2 (4%) had twin pregnancies. All 180 women who were enrolled were randomized to either cervical ripening with a combination of extra-amniotic Foley’s catheter and misoprostol (*n* = 90) or 25 micrograms of misoprostol alone (*n* = 90). There were no withdrawals and all participants received the assigned treatment.

There were no differences in the socio-demographic characteristics of women between the two groups (Table 1). The mean (standard deviation, SD) age of women in the combined misoprostol and extra-amniotic Foley’s catheter group was 27.8 (5.9) years compared to 26.9 (4.7) years in the misoprostol alone group. Most women belonged to the age group 25–34 years, were married, had above primary education, and were employed. The obstetric characteristics were equally similar between the combined extra-amniotic Foley’s catheter and misoprostol and the misoprostol only groups (Table 1). More than half of the women were multigravida and at gestational age of early term or greater. In both arms, the most common indication for labor induction was term or postterm pregnancy, which accounted for more than half (58%) of the participants. Pre-induction mean (SD) Bishop’s score was similar between the two groups 2.8 (1.1) versus 2.1 (1.5) for the combined versus misoprostol alone respectively.

**Table 1:** Baseline demographic and obstetric characteristics of study participants

	Intervention	
	Combination (Extra-amniotic Foley’s catheter + Vaginal misoprostol +) *N* = 90	Vaginal misoprostol alone *N* = 90
	*n* (%) or mean (SD)	*n* (%) or mean (SD)
Maternal age		
17–24 years	25 (27.7)	30 (33.3)
25–34 years	51 (56.7)	51 (56.7)
≥ 35 years	14 (15.6)	9 (10.0)
Marital status		
Single	16 (17.8)	17 (18.9)
Married	70 (77.8)	72 (80.0)
Separated/ divorced	4 (4.4)	1 (1.1)
Education level		
Primary	23 (25.6)	15 (16.7)
Secondary	34 (37.8)	33 (36.7)
Post-secondary	33 (36.7)	42 (46.7)
Employment		
Employed	52 (57.8)	56 (62.2)
Unemployed	38 (44.2)	34 (37.8)
Parity		
Primigravida	35 (38.9)	42 (46.7)
Gestation (weeks)		
28	9 (10.0)	6 (6.7)
29–39	19 (21.1)	20 (22.2)
≥ 39	62 (68.9)	64 (71.1)
Indication for induction		
≥ Full term	54 (60)	51 (56.7)
Hypertension	12 (13)	17 (18.9)
Rhesus incompatibility	3 (3.3)	5 (5.5)
Intrauterine fetal demise	12 (13.3)	11 (12.2)
Others^a^	9 (10)	6 (6.7)
Bishops score	2.8 ± 1.1	2.1 ± 1.5

*SD* standard deviation, ^a^Others include reduced fetal movements

Overall, 18(10%) of the participants had failed induction, 10 (8.9%) in the combined extra-amniotic Foley’s catheter and misoprostol group and 8 (11.1%) in the misoprostol alone group (Table 2). There was no statistically significant difference in the risk of failed induction between the two arms. Pregnant women in the combined extra-amniotic Foley’s catheter and misoprostol group versus misoprostol group were less likely to receive a second (66.7% versus 88.9%, *p* < 0.001) or third (26.7% versus 46.7, *p* = 0.006) dose of misoprostol compared to those in the misoprostol alone group (Table 2). This reduced the need of a second and a third dose misoprostol by 25% (Relative Risk [RR] 0.75; 95% Confidence Interval (CI) 0.75(0.63–0.88) *p* < 0.001 and 68% (RR 0.57 (95% CI 0.22–0.78), *p* = 0.005 respectively. There were no differences in the need of administration of the fourth dose of misoprostol.

**Table 2:** Maternal, labor and neonatal outcomes following induction of labour with misoprostol and Foley versus misoprostol alone

	Combination (Extra-amniotic Foley’s catheter and Vaginal misoprostol) *N* = 90	Vaginal misoprostol alone *N* = 90	RR (95% CI)	*P* value
	*n* (%)	*n* (%)		
Failed induction				
No	82 (91.1)	80 (89.9)	1.0 (Reference)	
Yes	8 (8.9)	10 (11.1)	0.80 (0.33–1.93)	0.619
Number of doses of misoprostol administered				
One (*n* = 90)	90 (100)	90 (100)	1.0 (Reference)	
Two (*n* = 90)	60 (66.7)	80 (88.9)	0.75 (0.63–0.88)	< 0.001*
Three (*n* = 90)	24 (26.7)	42 (46.7)	0.57 (0.38–0.86)	0.005*
Four (*n* = 90)	8 (8.9)	16 (17.8)	0.50 (0.23–1.11)	0.079
Mode of delivery				
Vaginal	72 (80.0)	76 (84.4)	1.0 (Reference)	
Cesarean section	18 (20.0)	14 (15.6)	1.29 (0.68–2.42)	0.435
Maternal complications				
None	86 (95.6)	82 (91.1)	1.0 (Reference)	
Postpartum hemorrhage	3 (3.3)	4 (4.4)	0.75 (0.17–3.26)	0.707
Uterine hyper stimulation	1 (1.1)	3 (3.3)	0.33 (0.01–4.16)	0.315
Uterine rupture	0 (0.0)	1 (1.1)	NA	NA
Neonatal complications				
Admission to NICU	15 (16.7)	17 (18.9)	0.86 (0.37–1.98)	0.697
Five minute APGAR score < 7	7 (7.8)	6 (6.7)	1.18 (0.32–4.44)	0.773
Prematurity	8 (8.9)	11 (12.2)	1.43 (0.55–3.73)	0.468

*RR* relative risk *CI* confidence interval, *Statistically significant differences, *NICU* neonatal intensive care unit, *NA* not applicable

**Fig. 2:**
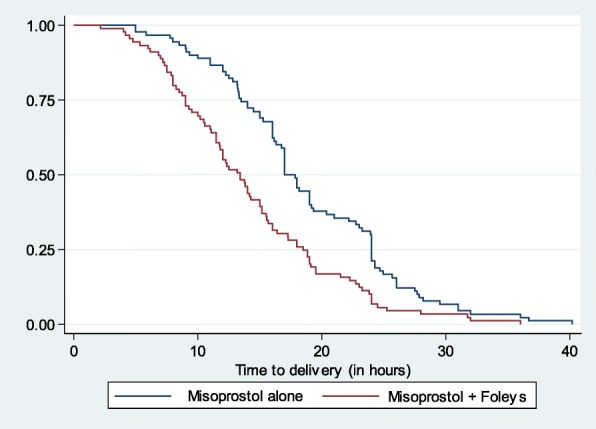
Kaplan–Meier time curve for induction-to-delivery interval. *Mean hours to delivery = 14.1 standard deviation 6.9 and 18.9 standard deviation (7.2) for extra-amniotic Foley’s catheter and misoprostol* versus *vaginal misoprostol alone. Mean difference = 4.8(2.7–6.8) hours, p < 0.001*

Women randomized to the combined extra-amniotic Foley’s catheter and misoprostol group had a significantly shorter duration of time between the onset of induction of labor and delivery (induction-delivery-time) compared to those in the misoprostol only group (Fig. 2). The Kaplan–Meier survival curve showed a statistically significantly steeper and different rate of decline in the extra-amniotic Foley’s catheter plus misoprostol group (log-rank test *P* < 0.001) compared to the misoprostol alone group. The mean induction-to-delivery interval was 4.8 h shorter comparing the combined versus misoprostol alone groups. The mean (standard deviation, (SD)) induction-to-delivery interval was 14.1 (6.9) hours for women in the combination of extra-amniotic Foley’s catheter and misoprostol compared to 18.9 (7.2) hours for those in the misoprostol alone (*p* < 0.001).

There were more maternal complications in the misoprostol alone arm (8.9%) compared to the combined misoprostol and extra-amniotic Foley’s catheter arm (4.4%) including one uterine rupture in the misoprostol only arm (Table 3). However, the complications were not statistically significantly different between the two study arms. Similar proportion of neonates in the combined misoprostol and extra-amniotic Foley’s catheter group compared to those in the misoprostol alone group were admitted to neonatal intensive care unit due to poor APGAR score (7.8% in combined arm versus 6.7% in misoprostol alone), and prematurity (9.8% in combined versus 12.2% in misoprostol alone) (Table 2).

## Discussion

In this randomized trial, a combination of extra-amniotic Foley’s catheter and vaginal misoprostol compared to vaginal misoprostol alone for cervical ripening and induction of labor did not statistically significantly reduce the rates of failed induction but shortened the induction-to-delivery interval by 4 h, reduced the number of doses of misoprostol administered, and there was no difference in risk of adverse maternal and early perinatal outcomes.

Our finding of no statistically significant reduction in the incidence of failed induction comparing combination of extra-amniotic Foley’s catheter and misoprostol versus misoprostol alone is consistent with prior studies [6, 7, 10, 11]. Across these studies, failed induction was consistently defined as failure to initiate labor with more than 3 cm cervical dilatation after 24 h of induction [14] as opposed to Baños who defined failed induction as inability to go into active labor after 36 h or failure to deliver within 12 h after active phase of labor [12]. This difference in definitions may account for some of the differences in rates of failed induction seen in different studies.

Induction of labor with combination of extra-amniotic Foley’s catheter plus vaginal misoprostol shortened the overall time from induction-to-delivery by 4.8 h in comparison to misoprostol alone in our study. Our results are consistent with at least three previous studies [6, 7, 14]. Carbone and colleagues found 3.1 h’ reduction in induction-to-delivery time when they compared combined extra-amniotic Foley’s catheter plus misoprostol versus misoprostol alone [6]. Dahiya reported that combined Foley and vaginal misoprostol shortened induction-to-delivery interval by 2.78 h while Levine reported 4.5 h reduction in induction-to-delivery interval comparing extra-amniotic Foley’s catheter plus vaginal misoprostol alone [7, 14]. Overall, these trials show that combined methods significantly shorten the induction-to-delivery time. The reduction in induction-to-delivery time could be due to the synergistic effect arising from the effect of prostaglandin on the cervix and the the Foley’s catheter dilation of the cervical coupled with local release of additional prostaglandins.

Regarding safety, there were no significant differences in the rates of adverse maternal and perinatal outcomes or complications in our study. This outcome is similar to findings from prior trials by Carbone who reported higher but non-significant rates of cesarean section in the combined compared to the misoprostol alone group [6]. Similarly, Dahiya and Levine did not find significant differences in adverse maternal and neonatal outcomes [7, 15].

Our study had several strengths. It was conducted in a resource limited setting making its findings generalizable to similar settings in low and middle income countries. Due to the short time between intervention and outcome, we had universal retention and adherence to the study protocol hence no post randomization bias. Also, we had study participants with varying indications for induction, at diverse gestational ages and varying parity making our results more representative and therefore generalizable to all women scheduled for induction of labor.

Despite these strengths, our study had some limitations. There was no blinding of investigators due to the nature of the study and this may have influenced patient management. However, since we did not have any dropouts or loss to follow up this may have not negatively affected our study. Our study was not powered to measure some of the secondary outcomes due to the large sample sizes, which would be required.

## Conclusion

This randomized clinical trial demonstrates that a combination of extra-amniotic Foley’s catheter and misoprostol for cervical ripening and induction of labor does not significantly reduce failed induction but shortens induction-to-delivery interval without increasing adverse maternal and early perinatal outcomes. Therefore, in clinical situations where induction-to-delivery interval is a priority, and Bishop’s score is poor, health care providers should consider offering combined extra-amniotic Foley’s catheter and misoprostol rather than misoprostol alone for cervical ripening and induction of labor. In reducing the duration of induction-to-delivery, these findings may reduce the cost of hospitalization and related maternal anxiety in this setting. Additional studies should also evaluate the cost-effectiveness, level of maternal satisfaction, and long term effects of combining extra-amniotic Foley’s Catheter and misoprostol versus misoprostol alone.

## Additional file

Additional file 1: Registered Protocol: A combination of foley baloon and misoprostol versus misoprostol alone for induction of labour at Kenyatta national hospital, a randomized controlled trial. (DOCX 84 kb)

## Abbreviations

APGAR: Appearance Pulse Grimace Activity and Respiration
CI: Confidence interval
DSMB: Data and Safety Monitoring Board
HIV: Human immunodeficiency virus
KNH: Kenyatta National Hospital
ORWH: Office of Research on Women’s Health
RR: Relative Risk
SD: Standard deviation
UoN: University of Nairobi
