# Supplementary material for: Effect of extra-amniotic Foley’s catheter and vaginal misoprostol versus vaginal misoprostol alone on cervical ripening and induction of labor in Kenya, a randomized controlled trial
Source: BMC Pregnancy Childbirth. 2018 Jul 12;18:300. doi: 10.1186/s12884-018-1793-2 (PMC6044072; doi:10.1186/s12884-018-1793-2)
Supplement: Supplementary file 1 — Registered Protocol: A combination of foley baloon and misoprostol versus misoprostol alone for induction of labour at Kenyatta national hospital, a randomized controlled trial. (DOCX 84 kb) [file 12884_2018_1793_MOESM1_ESM.docx]

A COMBINATION OF FOLEY BALOON AND MISOPROSTOL VERSUS MISOPROSTOL ALONE FOR INDUCTION OF LABOUR AT KENYATTA NATIONAL HOSPITAL, A RANDOMIZED CONTROLLED TRIAL

UNIVERSITY OF NAIROBI

DEPARTMENT OF OBSTETRICS AND GYNAECOLOGY

PRINCIPAL INVESTIGATOR:

ALFRED OSOTI, TITO TONG, DAVIES KIPROP KIBII, INNOCENT MARANGA, ALFRED OSOTI.

**LIST OF ABBREVIATIONS**

ACOG – American College of Obstetricians and Gynecology

WHO– World Health Organization.

NICU – Neonatal Intensive Care Unit.

GAGs– Glycosaminoglycan.

RCT– Randomized Clinical Trial.

IUFD– Intrauterine Fetal Demise.

ICU – Intensive Care Unit.

IUGR – Intrauterine Growth Restriction.

KNH – Kenyatta National Hospital.

USA– United States of America.

**ABSTRACT**

The World Health Organization (WHO) defines induction of labor as the initiation of labor by artificial means prior to its spontaneous onset at a viable gestational age with the aim of achieving vaginal delivery in a pregnant woman with intact membranes. Successful labor induction leads to vaginal birth while failed induction is the inability to achieve more than 3 cm cervical dilatation after 24 hours of induction of labor. In developed countries, up to 25% of term deliveries involve induction of labor compared to about 9.6% in the developing countries (6). Failed induction is an expected outcome of induction of labor. Combination methods of induction of labor may reduce the high failed induction rates reported by misoprostol or Foley catheter balloon alone.

In this study we propose a randomized trial whose aim is to find out if combined methods have a higher success rate.one arm will utilize misoprostol alone and the other combined misoprostol with Foley’s catheter. The outcomes will include successful induction, maternal and early neonatal outcomes. Failed induction or prolonged duration of induction to delivery may increase costs, patient anxiety, and if not monitored well especially in the setting of heavy workload may lead to poor neonatal outcomes.

If induction of labor using combined Foley and misoprostol can reverse or reduce these outcomes then it can change guidelines on induction of labor or for those patients at risk of failed induction.

A similar study has also not been carried out at our study setting.

**Introduction**

INDUCTION OF LABOUR

Induction of labor is a procedure used to stimulate uterine contractions during pregnancy before spontaneous labor. Successful labor induction leads to a vaginal birth. A health care provider might recommend labor induction for various indications, primarily when there's concern for a mother's health or a baby's health. In developed countries, up to 25% of all deliveries at term now involve induction of labor. In developing countries, the rates are generally lower, but in some settings they can be as high as those observed in developed countries,(World Health Organization, WHO(3)

Induction of labor is not risk-free and many women find it to be uncomfortable. With a view to promoting the best known clinical practices in labor and childbirth and to improving maternal outcomes worldwide, WHO has developed the present recommendations using the procedures outlined in the *WHO Handbook for guideline development(3)*

General principles related to the practice of induction of labor

Induction of labor should be performed only when there is a clear medical indication for it and the expected benefits outweigh its potential harms.

In applying the recommendations, consideration must be given to the actual condition, wishes and preferences of each woman, with emphasis being placed on cervical status, the specific method of induction of labor and associated conditions such as parity and rupture of membranes.

Induction of labor should be performed with caution since the procedure carries the risk of uterine hyper stimulation and rupture and fetal distress.

Wherever induction of labor is carried out, facilities should be available for assessing maternal and fetal well-being.

Women receiving oxytocin, misoprostol or other prostaglandins should never be left unattended.

Failed induction of labor does not necessarily indicate caesarean section.

Wherever possible, induction of labor should be carried out in facilities where caesarean section can be performed.

The indication for induction of labor therefore must be convincing, compelling, consented to and documented. These conditions are not met when the proposed indication is solely for the convenience of the physician or the woman alone.

Specific recommendations for induction of labor

1. Induction of labor is recommended for women who are known with certainty to have reached 41 weeks (>40 weeks + 7 days) of gestation.
2. Induction of labor is not recommended in women with an uncomplicated pregnancy at gestational age less than 41 weeks.
3. If gestational diabetes is the only abnormality, induction of labor before 41 weeks of gestation is not recommended.
4. Induction of labor at term is not recommended for suspected fetal macrosomia.
5. Induction of labor is recommended for women with prelabour rupture of membranes at term.
6. For induction of labor in women with an uncomplicated twin pregnancy at or near term, no recommendation was made as there was insufficient evidence to issue a recommendation. If prostaglandins are not available, intravenous oxytocin alone should be used for induction of labor. Amniotomy alone is not recommended for induction of labor.

Methods of induction of labor

Methods of labor induction include mechanical and pharmacological means. Optimal choice of these depends on the pre-induction status of the cervix. The three factors most likely to lead to success include favorable cervix, multiparity and prior vaginal delivery. The cervix is considered unfavorable if the bishop score is less than 6.The most important element of bishop score is dilation, followed by effacement, station and position, with the least useful element being cervical consistency. Xenakis et al clearly demonstrated that women who had a bishop score of 3 or less at onset of induction had significantly higher rates of failed induction and caesarean delivery then those with a bishop score above 3.

Table 1-Bishop scoring system.

| Factor | 0 | 1 | 2 | 3 |
| --- | --- | --- | --- | --- |
| Dilatation (cm) | 0 | 1-2 | 3-4 | >5 |
| Effacement (%) | 0-30 | 40-50 | 60-70 | >80 |
| Consistency | Firm | Medium | Soft |  |
| Position | Posterior | Central | Anterior |  |
| Station | -3 | -2 | -1 or 0 | +1 or lower |

WHO has proposed the following recommendations,

1-If prostaglandins are not available, intravenous oxytocin alone should be used for induction of labor. Amniotomy alone is not recommended for induction of labor.

2- Oral misoprostol (25 μg, 2-hourly) is recommended for induction of labor.

3- Low-dose vaginal misoprostol (25 μg, 6-hourly) is recommended for induction of labor.

4- Misoprostol is not recommended for induction of labor in women with previous caesarean section.

5- Low doses of vaginal prostaglandins are recommended for induction of labor.

6-Balloon catheter is recommended for induction of labor.

7-The combination of balloon catheter plus oxytocin is recommended as an alternative method of induction of labor when prostaglandins (including misoprostol) are not available or are contraindicated.

8-In the third trimester, in women with a dead or an anomalous fetus, oral or vaginal misoprostol are recommended for induction of labor.

9- Sweeping membranes are recommended for reducing formal induction of labor.

MANAGEMENT OF ADVERSE EFFECTS ASSOCIATED WITH INDUCTION OF LABOUR

Betamimetics are recommended for women with uterine hyper- stimulation during induction of labor. Uterine hyper stimulation is defined as either occurrence of uterine contractions lasting more than 60 seconds, or occurrence of more than four contractions within 10 minutes, regardless the state of the fetus.Terbutaline is the main beta mimetic used.Tocolytics like magnesium sulfate,atosiban and nitroglycerin have a small effect(3).

Setting for induction of labor

Outpatient induction of labor is not recommended for improving birth outcomes. This because induction of labor requires facilities for monitoring maternal and fetal well-being. Secondly induction should be done in a facility which has the capacity of doing a cesarean section(3).

**DEFINITION OF FAILED INDUCTION**.

Failed induction is defined as labor not starting after one cycle of treatment or after 24 hours. If induction fails, healthcare professionals should discuss this with the woman and provide support. The woman’s condition and the pregnancy in general should be fully reassessed, and fetal wellbeing should be assessed using electronic fetal monitoring. If induction fails, decisions about further management should be made in accordance with the woman’s wishes, and should take into account the clinical circumstances. The subsequent management options include: a further attempt to induce labor (the timing should depend on the clinical situation and the woman’s wishes) or caesarean section.(7). A study done by Osaheni Lucky et al. (Nigeria, 2014) found out 24.1% rate of failed induction. Locally, a study done by Esiromo (KNH, 2011) found out 26% rates of failed induction. The study also found out 77.7% success rate with misoprostol alone and 40% success with Foleys catheter alone.

**Predictors of successful induction of labor**.

Successful induction of labor is more likely if there is a good Bishop score, more than 3,multiparity and Prior vaginal delivery. Xenakis et.al clearly demonstrated that women who had a bishop score of 3 or less at onset of induction had significantly higher rates of failed induction and caesarean delivery then those with a bishop score above 3.

**CONCEPTUAL FRAMEWORK**

Women for induction of labour

Those recruited and allocated the combination arm

Those recruited and allocated misoprostol arm

Misoprostol and Foley catheter administered as per protocol

Misoprostol administered as per protocol

Increased intracellular calcium,elastase and GAGs

Increased intracellular calcium,elastase and glycosaminoglycan.local pressure effect of the balloon on the cervix

Cervical dilatation and onset of labour.

Faster cervical dilatation of the cervix and shorter duration of labour.

**SCHEMA OF TRIAL**

**Screened**

**N=**

**Enrolled**

**N=**

**Foley catheter and misoprostol**

**N=**

**Misoprostol**

**N=**

**Primary outcome: failed induction**

**N=**

**Primary outcome: failed induction**

**N=**

**Secondary outcomes**

**N=**

**Secondary outcomes**

**N=**

**Study justification**

Failed induction or prolonged duration of induction to delivery may increase costs, patient anxiety, and if not monitored well especially in the setting of heavy workload may lead to poor neonatal outcomes.

If induction of labor using combined Foley and misoprostol can reverse or reduce these outcomes then it can change guidelines on induction of labor or for those patients at risk of failed induction.

A similar study has also not been carried out at our study setting.

**Research question**

Is there a difference in the incidence of failed induction if combined Foley catheter plus misoprostol versus misoprostol alone is used for cervical ripening and induction of labor?

**Hypothesis**

There is no difference in the incidence of failed induction when combined Foley catheter plus misoprostol versus misoprostol alone is used cervical ripening and induction of labor.

**OBJECTIVES.**

**BROAD OBJECTIVE:**

To evaluate the efficacy of combined mechanical (Foley balloon) and pharmacologic (misoprostol) administration in reducing the incidence of failed induction.

**SPECIFIC OBJECTIVES**

Among pregnant women undergoing cervical ripening and induction of labor using combined misoprostol and Foley balloon versus misoprostol alone,

1. Determine and compare the incidence of failed induction.
2. Determine and compare the induction to delivery time.
3. Determine and compare immediate maternal and perinatal outcomes.

**METHODOLOGY**

**Study design**

Two arm open label (non blind) RCT

**Study population**

Pregnant women with Bishop’s score of 6 or less presenting to KNH labor wards for labor induction

**Intervention arm**

Foley plus misoprostol

**Control arm**

Misoprostol alone

**Outcomes**

Primary outcome-failed induction

**Secondary outcome**

Induction to delivery time

Mode of delivery

Maternal outcomes-PPH, chorioamnionitis,hyperstimulation(5 contractions in 5mins)

Perinatal outcomes (APGAR, NICU admission

Methodology

Block randomization of all eligible consenting subjects by computer generated random sequences and a randomization ratio of 1:1.Upon enrollment, an opaque envelope containing the participant’s enrollment number and assignment to either the Foley plus vaginal misoprostol or vaginal misoprostol alone will be opened .Each participant will be assigned a unique 4-digit-long subject number for subject identity and confidentiality. Participants and health provider will be aware of the treatment allocation at the time of assignment of treatment.

Study procedures.

Patients who fall in the Misoprostol arm will be given 25 mcg vaginally 6hourly up to a maximum of 4 doses, Bishop score >6 or when they go into active labor. Later amniotomy and augmentation may be done as per protocol

Patients who fall in the Combination arm will be inserted an 18-French Foley catheter with a 30 cc balloon will be placed just above the internal cervical os and then inflated with 30 cc of sterile saline. The length of the Foley catheter will be affixed to the subject’s inner thigh under slight tension. At the same time 25 mcg of misoprostol is inserted at the posterior fornix of the vagina 6 hourly up to a maximum of 4 doses, Bishop score >6 or active labor. If difficult, Foley catheter insertion will be attempted every 6 hours unless Bishop score more than 6 or in active labor. When Foley balloon falls off, labor may be augmented or amniotomy performed as per the existing protocol.

**Study setting.**

This study is to be carried out at the Kenyatta Hospital (KNH) antenatal and labor wars. KNH is the largest teaching and referral hospital in Kenya. It receives patients from Nairobi and its environs as well as referrals from all other hospitals in Kenya. It has a bed capacity of 1800 beds and is located 2km southwest of the Nairobi Central Business District.

Mothers will be recruited as they are admitted for delivery due to an indication that warrants induction of labor (WHO) which include

1. Postdates
2. Pre eclampsia.
3. Chronic hypertension.
4. Gestational diabetes.
5. Oligohydramnios.
6. Intrauterine Fetal Demise.

**Study population**

Pregnant women admitted for induction of labor at KNH at gestational age of 28 weeks and beyond.

INCLUSION & EXCLUSION CRITERIA:

Inclusion:

Intrauterine gestation, cephalic presentation, intact membranes, IUFD and Bishop score less than 6.

Willing to give informed consent.

Exclusion:

IUGR, Previous cesarean sections, multiple gestation, contraindication to prostaglandins, fetal anomalies, other uterine surgeries, EFW>4000G,Placenta previa, NRFS, multiparity >5,HIV infected and those not sure of dates by clinical examination as well as ultrasound dating.

**Sample size calculation**

A recent Kenyan study placed failed induction at 26%.

We postulate that offering a combined use of misoprostol and Foley catheter will reduce this proportion to 16%.

Therefore for us to detect a 16% difference in the successful induction following the use of Foley with misoprostol versus misoprostol alone, we estimated using the sample size formula,

Sample size formula$n=\frac{2\left(z_{1-\alpha_{/2}}\sqrt{2\bar{p}(1-\bar{p})}+z_{1-\beta}\sqrt{p_{c}(1-p_{c})+p_{a}(1-p_{a})}\right)^{2}}{(p_{c}-p_{a})^{2}}$

[Allan Donner; Stat. Medicine (1984)

that we would need to study a total of 180 women (90 per group) to achieve a 80% power to detect the stated difference of 16% at a two-sided alpha=0.05 level of significance.

Where we define p_c_=74% and p_a_=90% to be the proportions of women in the misoprostol and combined misoprostol and Foley groups respectively

and = (p_C_ + p_a_)/2 ( =1.960, and =0.84

**Data collection, management and analysis**.

Data collection and storage: Data will be collected by use of questionnaires by the principal investigator and 3 or 4 well trained research assistants. Information will be obtained from history, review of medical records and clinical examination. The information will be stored safely in a password-protected computer and backed up on a dedicated USB drive. Any hard copy records carried for analysis will be stored under lock and key and patients confidentiality will be observed. A qualified statistician will do analysis as per protocol with intent to treat analysis using SPSS version 21.Primary outcome will be failed induction/successful induction and secondary outcomes include time to delivery, maternal and perinatal outcomes. Proportions will be compares using Chi2 test, 95% CI, two tailed hypothesis with p significant at <0.05

Ethical considerations.

- Permission will be sought from the KNH/UON ERC.
- Informed Consent.
- Confidentiality.
- Patients who decline to give consent will be treated as per the existing protocols and no penalty at all.

Study limitations

Non-blinding may have influence patient management

No long-term follow up of mother and neonate for late outcomes

No interim analysis due to resources.

Study timelines

- Proposal presentation-January 2014
- ERC approval and registration-February 2015
- Enrollment-march-September 2015
- Analysis-September-December 2015
- Presentation-December 2015
- Publishing-December-June 2016
- Dissemination of results-June 2016

Study budget

The anticipated expenses during this study include the following-

Buying of Foleys catheters and misoprostol-

Stipend for the research assistants and the statistician.

Printing of the proposal, questionnaires and other paper work.

References.

1. Carbone et.al-combination of Foley with vaginal misoprostol compared with vaginal misoprostol alone for ripening and labor induction.
2. Moraes et.al-RCT comparing vaginal misoprostol versus Foley catheter plus oxytocin for labor induction.
3. WHO recommendations for induction of labor (2011)
4. Esiromo, (2011)
5. Donner, A., Approaches to sample size estimation in the design of clinical trials--a review. Stat Med, 1984. 3(3): p. 199-214.
6. WHO Global Survey on Maternal and Perinatal Health. Induction of labour data. Geneva; World health Organization: 2010.
7. NICE clinical guideline (2008)
